# Readout Circuits for Capacitive Sensors

**DOI:** 10.3390/mi12080960

**Published:** 2021-08-13

**Authors:** Yongsang Yoo, Byong-Deok Choi

**Affiliations:** Department of Electronic Engineering, Hanyang University, Seoul 04763, Korea; yongsangyoo@hanyang.ac.kr

**Keywords:** capacitive sensors, capacitive readout system, self-capacitance readout circuit, mutual capacitance readout circuit, signal power degradation, noise reduction, charge-balancing compensation, parasitic capacitance, chopper stabilization, (microelectromechanical systems) MEMS microphone readout circuit

## Abstract

The development of microelectromechanical system (MEMS) processes enables the integration of capacitive sensors into silicon integrated circuits. These sensors have been gaining considerable attention as a solution for mobile and internet of things (IoT) devices because of their low power consumption. In this study, we introduce the operating principle of representative capacitive sensors and discuss the major technical challenges, solutions, and future tasks for a capacitive readout system. The signal-to-noise ratio (SNR) is the most important performance parameter for a sensor system that measures changes in physical quantities; in addition, power consumption is another important factor because of the characteristics of mobile and IoT devices. Signal power degradation and noise, which degrade the SNR in the sensor readout system, are analyzed; circuit design approaches for degradation prevention are discussed. Further, we discuss the previous efforts and existing studies that focus on low power consumption. We present detailed circuit techniques and illustrate their effectiveness in suppressing signal power degradation and achieving lower noise levels via application to a design example of an actual MEMS microphone readout system.

## 1. Introduction

Recent growth in mobile devices and the internet of things (IoT) market has led to an increased demand for various types of sensors [1]. Mobile and IoT devices have a small size and are equipped with batteries; further, because of their size limitations, a small form factor is required for the sensor. Low power consumption is important for battery-powered devices that cannot operate for longer periods with their smaller batteries [2]. The electrical characteristics of the sensor change according to the physical quantity to be measured. Thus, depending on the type of change in the electrical property being measured, sensors can be classified as resistive, capacitive, inductive, and magnetic [3]. Among these, capacitive sensors have received considerable research attention because of the recent development of the microelectromechanical system (MEMS) processes [4,5,6]. Further, capacitive sensors have become a popular research topic as they are advantageous because of their small form factor and low power consumption [5,6,7].

Resistive, inductive, and magnetic sensors have static current paths, which inevitably consume power when operating sensors. The direct current(DC) does not flow because there is no DC current path in the capacitive sensor; this is advantageous in terms of power consumption. Some capacitive sensors require a high-voltage DC bias; however, even in these cases, there is no DC current flow. It is possible to decrease the power consumption of the sensor using various driving methods such as charge–discharge cycle adjustment or charge-sharing techniques because the power consumption of a capacitive sensor is determined by the charge–discharge charge, formulated as *Q = C* · *V*. Sensors that require mechanical movement among conventional capacitors (gyroscope, accelerator, pressure sensor, etc.) are difficult to implement in small sizes because of problems such as the production, assembly, and durability of mechanical parts [7]. The recent development of MEMS processes has facilitated the development of capacitive sensors with mechanical movement to be implemented on a very small scale on a semiconductor chip, which meets the needs of the small form factor.

A capacitive sensor system comprises a capacitive sensor and readout system. The readout system and sensor are very important in terms of the overall system performance and power consumption. Although the capacitive sensor has few noise sources, most of the noise is introduced from the outside or from the other major circuits of the readout system. Further, because the power consumption of the capacitive sensor is low, the power consumptions of other circuits (analog front-end circuit, analog-to-digital converter, post-processor, etc.) of the readout system are dominant. Therefore, most major specifications, such as the signal-to-noise ratio(SNR) and power consumption required for a capacitive sensor system, are more affected by the readout system than the sensor itself. Thus, the capacitive readout system is very important for achieving specific goals such as meeting SNR or power consumption requirements. Among the capacitive readout systems, the analog front-end circuitry, which is the first of the readout systems to be connected to the sensors, uses different structures and driving methods based on the characteristics of the sensor; further, it has a significant influence on the overall performance of the entire readout system. That is, the effective performances of other circuits such as analog-to-digital converters (ADCs) and post-processing logic circuits that receive signals from the analog front-end circuit and the design target specifications considerably depend on the performance of the analog front-end circuit.

The rest of this paper is organized as follows. The different types of capacitive sensors and the structure of the basic readout system are discussed in Section 2. The main technical issues that degrade the performance of the capacitive readout system are presented in Section 3, in addition to the methods employed to resolve these issues. Finally, in Section 4, we introduce the readout circuit design method through an actual design example of the analog front-end circuit for the MEMS microphone.

## 2. Capacitive Sensor and Readout System

**Figure 1:**
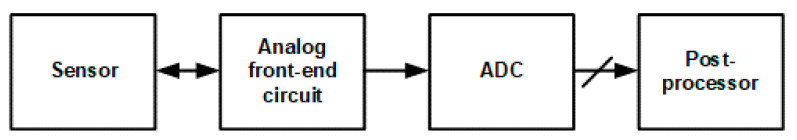
Block diagram of capacitive sensor and readout system.

In this section, the basic configuration and features of the capacitive sensor and its readout system will be introduced. The typical configuration of a capacitive sensor readout system is shown in Figure 1. The system comprises an analog front-end circuit, which interfaces with the sensor and converts the capacitance change caused by the change in the physical quantity to be measured into voltage or current; an ADC, which converts analog signals to digital signals; and a post-processor for signal post-processing. Based on the application, the sensor and ADC can be directly connected except for the analog front-end circuit; if the output is analog, it may only comprise the sensor and the analog front-end circuit.

### 2.1. Capacitive Sensor

There are various types of capacitive sensors; however, for the convenience of understanding, if we model the capacitance between two flat electrodes (*C =* *εA/d)*; the capacitive sensors can measure the permittivity, the distance between the two electrodes, or the area change caused by the change in the physical quantity to be measured; the capacitive sensors can be classified based on these changes.

**Figure 2:**
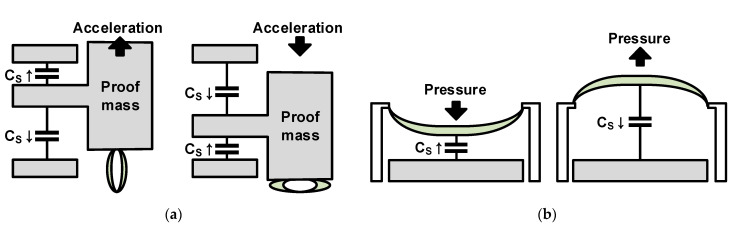
Distance-based capacitive sensors: (**a**) accelerometer; (**b**) pressure sensor.

An accelerometer, gyroscope, pressure sensor, and microphone are representative capacitive sensors based on the change in distance between the two electrodes. These sensors are manufactured through the MEMS process, which allows the mechanical movement of the electrode via the addition of the membranes to the IC fabrication process. A fixed electrode and membrane, which is a movable electrode, forms a capacitor in the sensor. The distance between the two electrodes is changed by a physical force (accelerating force, pressure, etc.), and when the physical force is reduced, the distance returns to the original value because of the elastic force of the membrane. As examples of sensors that follow this principle, the accelerometer and the pressure sensor are shown in Figure 2a,b. In Figure 2, the gray and green parts represent the electrode and the membrane, respectively. For signal amplitude and linearity, some sensors need the application of a DC bias between the electrodes of the sensor to generate an electrostatic force, as the membrane needs to exhibit appropriate elastic force.

**Figure 3:**
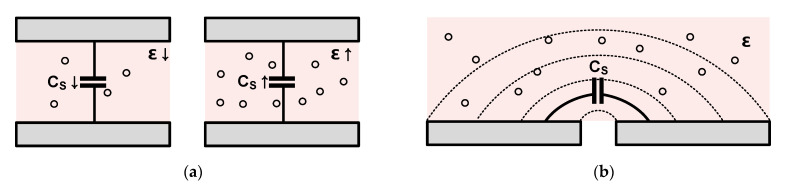
Permittivity-based capacitive sensors: (**a**) vertically placed concentration sensor; (**b**) horizontally placed concentration sensor.

Capacitive sensors based on changes in permittivity between two electrodes include a humidity sensor, particle concentration sensor, and chemical and biomedical substance concentration sensors. As shown in Figure 3a, a capacitive sensor has a fixed sensor electrode; the electrode can be arranged horizontally, as shown in Figure 3b. Because there is no physical movement, no special process such as the MEMS process is required, and it can be manufactured relatively easily via the IC process without the need for bias voltage. In addition, a fast sensing speed is not required because the concentration of the material to be measured by these sensors does not demonstrate abrupt changes easily. In addition to the above-mentioned method for detecting changes in the distance and permittivity between two electrodes, a displacement and position sensor is used to measure the capacitance change caused by the change in the overlapping area between the electrodes depending on the horizontal movement between the electrodes of the two objects.

**Figure 4:**
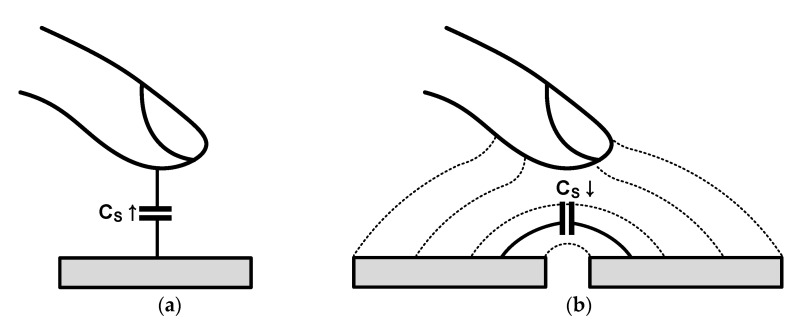
Capacitive touch sensors: (**a**) self-capacitive type; (**b**) mutual-capacitive type.

Most capacitive sensors are configured either alone or in pairs for the differential. Because the touch sensor is used in the form of an array on a display panel with a large physical size, there is a considerable difference in the environment from other capacitive sensors. In other sensors, the noise of the readout circuit is the dominant noise source. In the touch sensor, the display noise becomes the most dominant noise source because the touch and display panels are very close to each other, which results in a large parasitic capacitance between each other. Further, for this same reason, the noise from outside the sensor (such as charger noise and human noise) is a significant noise source. The required report rate in a touch application is lower than that of the other sensors; however, the sensing time per sensor is shorter than that of other sensors because the sensor is in the form of an array. The time available for sensing per sensor is very limited because touch sensing is only performed during the idle period of the display driving to cope with the display noise. Further, the touch sensor can be divided into self-capacitive and mutual-capacitive types, as shown in Figure 4a,b, respectively, according to the sensor structure and the principle of sensing capacitance.

For the self-capacitive type sensor, the finger is sensed using capacitance change caused by the distance between the finger and the touch electrode. Because all capacitances generated from a single sensing electrode (the point at which touch is to be sensed) are measured, the parasitic capacitance is very large in the case of a large-area touch panel, which is disadvantageous in that the signal power caused by the touch is not sufficiently large. The mutual-capacitive type sensor senses changes in capacitance between the two electrodes caused by fringe field interference. The mutual-capacitive type does not suffer from signal power degradation caused by parasitic capacitance as in the self-capacitive type; however, because the fringe field between the two electrodes is used, one disadvantage is that the amount of change in capacitance is rapidly reduced when the distance between the finger and electrodes in the touch sensor increases as the covering glass becomes thicker. This sensor does not suffer from a lack of signal power such as that in the self-capacitive type sensor; however, the use of the fringe field between the two electrodes causes the amount of change in capacitance to rapidly become insignificant owing to the distance between the finger and the electrodes.

### 2.2. Analog Front-End Circuit

An analog front-end circuit can be classified into a circuit that reads the self-capacitance and another that reads the mutual capacitance. The former measures all capacitances occurring in one node connected to the sensor, and the latter measures the capacitance between the two separated connections with a connection to drive the sensor and a connection for measuring the sensor.

#### 2.2.1. Self-Capacitance Readout Circuit

**Figure 5:**
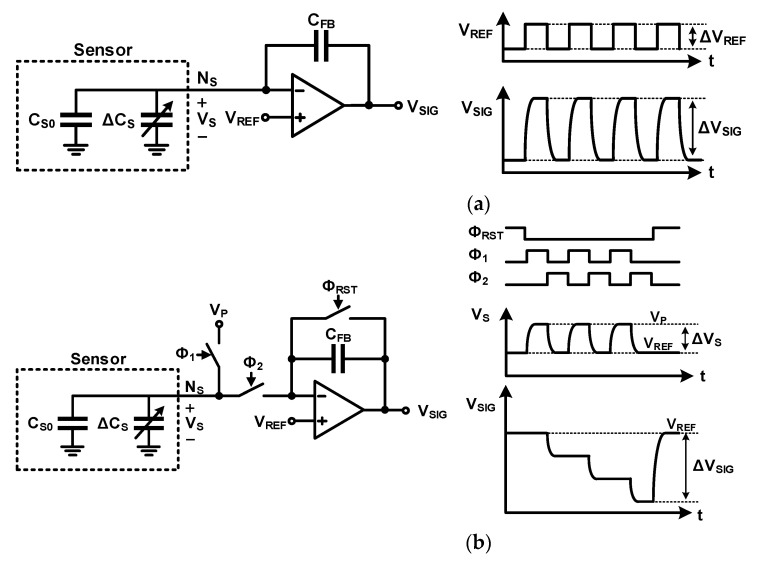
Readout circuits for self-capacitance sensing: (**a**) using charge amplifier; (**b**) using charge integrator.

The most common method of sensing self-capacitance is measuring the amount of charge required to charge the sensor. The circuit shown in Figure 5a is an example of a self-capacitance readout circuit that uses a charge amplifier. The node where the readout circuit and sensor are connected (*N_S_*) is virtually shorted with the reference node (*N_REF_*) by the charge amplifier. The charge amplifier charges and discharges the sensor such that the voltage across the sensor (*V_S_*) is equal to the voltage applied to the *N_REF_* (*V_REF_*). The charge required to charge and discharge the sensor is provided via a feedback capacitor (*C_FB_*) by the change in the output signal (Δ*V_SIG_*). Δ*V_REF_* is added together with the voltage change required for sensor charging and discharging to Δ*V_SIG_*. Δ*V_SIG_* is obtained as

(1) $$\begin{array}{cc} \Delta V_{SIG} & =\Delta V_{REF}+\frac{\left(C_{S0}+\Delta C_{S}\right)\left(\Delta V_{S}=\Delta V_{REF}\right)}{CF_{B}}\;\\ & =\left(1+\frac{C_{S0}+\Delta C_{S}}{CF_{B}}\right)\Delta V_{REF} \end{array}$$

As shown in Equation (2), the change in the self-capacitance (Δ*C_S_*) can be calculated from Δ*V_SIG_* as

(2) $$\Delta C_{S}=\left(\frac{\Delta V_{SIG}}{\Delta V_{REF}}-1\right)C_{FB}-C_{S0}$$

In contrast, as shown in the circuit in Figure 5b that uses the switch-capacitor charge integrator, the pre-charge and measurement phases are separated through the discrete-time operation. Further, there is an advantage that signals can be easily accumulated by repeating the pre-charge and measurement phases without resetting the stored charge in the *C_FB_*. The sensor capacitor charged to the pre-charge voltage (*V_P_*) during phase 1 (*Φ*_1_) is discharged to *V_REF_* during phase 2 (*Φ*_2_). The amount of charge required to discharge the sensor capacitor is provided by the *C_FB_*. After that, if phases 1 and 2 are repeated without resetting the stored charge in the *C_FB_*, the amount of charge sensed in the *C_FB_* is accumulated, and a large signal is obtained. If the phase 1–2 operation is repeated N times, Δ*V_SIG_* and Δ*C_S_* are obtained as

(3) $$\Delta V_{SIG}=-N\frac{C_{S0}+\Delta C_{S}}{C_{FB}}\left(V_{P}-V_{REF}\right)$$

(4) $$\Delta C_{S}=-\frac{\Delta V_{SIG}}{N\left(V_{P}-V_{REF}\right)}C_{FB}-C_{S0}$$

**Figure 6:**
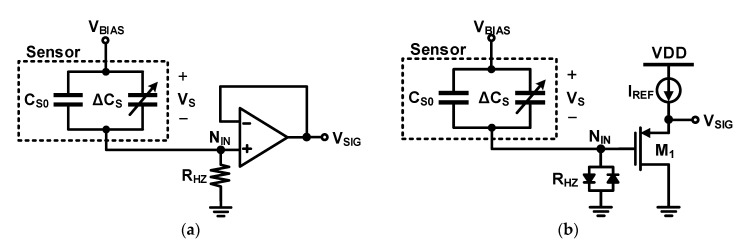
Direct current (DC) biased self-capacitance readout circuit: (**a**) using resistor and buffer; (**b**) using back-to-back diode and source follower.

The above-mentioned circuits measure the capacitance of the sensor based on the principle of detecting the amount of charge required to charge or discharge the sensor capacitor. The voltage difference between the charging and discharging of the sensor capacitor should be large to increase the output signal; therefore, the voltage applied to the sensor (*V_S_*) changes significantly during measurement. However, some sensors require a fixed bias voltage, and in this case, a readout circuit, as shown in Figure 6a, can be used instead of the circuits shown in Figure 5. In this circuit, a high-impedance path is formed at the node where the sensor and readout circuit are connected (*N_IN_*) to apply the DC bias of the node; however, the movement of the charge is prevented. Thus, the bias voltage of the capacitor is maintained, and the amount of charge in the capacitor does not change. If the capacitance changes because of the physical quantity change to be measured, the voltage changes as

(5) $$\begin{array}{ll} \Delta Q & =\Delta \left(C_{S}\right)\cdot V_{S}+C_{S}\Delta V_{S}\\ & =0 \end{array}$$

Further, the voltage at node N_IN_ changes as

(6) $$\begin{array}{ll} \Delta V_{IN} & =-\Delta V_{S}\\ & =\frac{\Delta C_{S}}{C_{S}}V_{BIAS}\\ & =\frac{\Delta C_{S}}{C_{S0}+\Delta C_{S}}V_{BIAS} \end{array}$$

In the case of a MEMS microphone, an appropriate elastic force must be generated in the membrane to exploit the performance of the sensor; to this end, a relatively high bias voltage must be constantly applied to the sensor. Figure 6b shows an example of a circuit widely used in microphone applications. A back-to-back diode is used to realize a high impedance DC bias, and a p-channel transistor source follower is used to receive an AC voltage signal with a ground DC voltage. This increases the DC level and lowers the impedance of the analog front-end circuit output so that it can match the input voltage range of the ADC or the output buffer that receives the signal from the source follower.

#### 2.2.2. Mutual-Capacitance Readout Circuit

**Figure 7:**
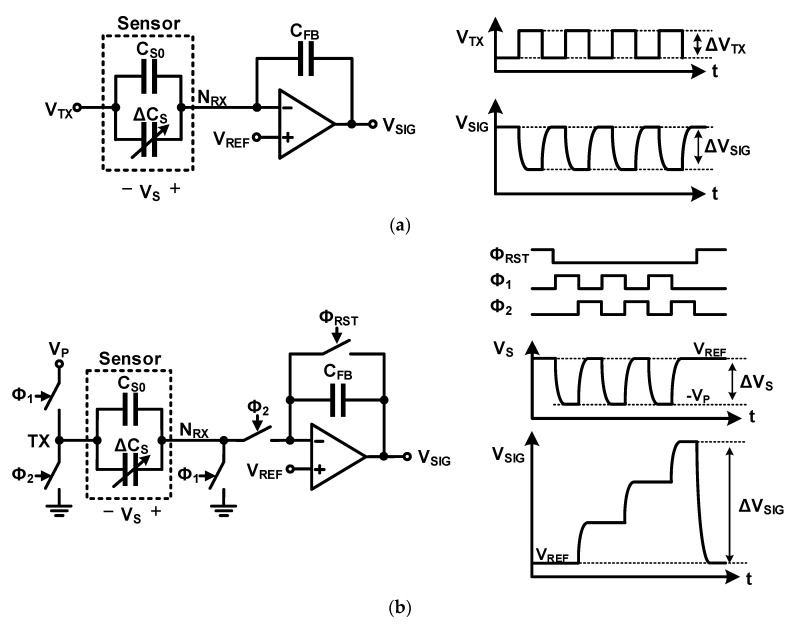
Readout circuits for mutual-capacitance sensing: (**a**) using charge amplifier; (**b**) using charge integrator.

A mutual capacitance readout circuit measures the capacitance between the electrodes of the sensor capacitor. It is different from the self-capacitance readout circuit in that the nodes that apply the charge–discharge voltage (*N_TX_*) and measure the change in the amount of charge generated (*N_RX_*) are separated. The simplest method to sense mutual capacitance is using a charge amplifier, as shown in Figure 7a. When a voltage change is applied to node *N_TX_* (Δ*V_TX_*), *V_S_* changes by −Δ*V_TX_*; a change in the amount of charge is required to charge and discharge the sensor capacitor. This charge is provided by the *C_FB_* via Δ*V_SIG_*. The generated Δ*V_SIG_* is given by

(7) $$\Delta V_{SIG}=-\frac{C_{S0}+\Delta C_{S}}{C_{FB}}\Delta V_{TX}$$

The capacitance of the sensor can be calculated using

(8) $$\Delta C_{S}=-\frac{\Delta V_{SIG}}{\Delta V_{TX}}C_{FB}-C_{S0}$$

If Δ*V_SIG_* is not sufficiently large, the output signal can be accumulated using a charge integrator composed of a switch capacitor, as shown in Figure 7b. Unlike the self-capacitance readout circuit shown in Figure 5b, the mutual capacitance readout circuit can increase the pre-charged voltage by changing the voltage of node *N_RX_* (*V_RX_*) in *Φ*_1_ because nodes *N_TX_* and *N_RX_* are separated. During *Φ*_1_, *V_S_* is charged to −*V_P_*, and during *Φ*_2_, it is discharged to *V_REF_*. While *V_S_* changes −*(V_P_ + V_REF_)* during *Φ*_1_ and *Φ*_2_, the self-capacitance readout circuit only changes *V_P_ − V_REF_* in the same process. When the number of repetitions of *Φ*_1_ and *Φ*_2_ is *N*, Δ*V_SIG_* and the measured capacitance are, respectively given as

(9) $$\begin{array}{ll} \Delta V_{SIG} & =-N\frac{C_{S0}+\Delta C_{S}}{C_{FB}}\left(-V_{P}-V_{REF}\right)\;\\ & =N\frac{C_{S0}+\Delta C_{S}}{C_{FB}}\left(V_{P}+V_{REF}\right) \end{array}$$

(10) $$\Delta C_{S}=\frac{\Delta V_{SIG}}{N\left(V_{P}+V_{REF}\right)}C_{FB}-C_{S0}$$

With the above method, noise reduction can be performed by arranging a filter to remove noise after changing the capacitance of the sensor into the form of voltage, current, etc. Subsequently, the signal is applied to the ADC, or if the analog signal is directly output to the outside; the output buffer is used as required.

### 2.3. Analog-to-Digital Converters (ADC)

The current or voltage signal from the analog front-end circuit is an analog signal with various levels. The current or voltage signal read from the sensor requires post-processing to improve the overall sensor readout system performance; most of this post-processing is performed in the digital domain. Therefore, the sensor readout system often includes an ADC that converts the analog output of the analog front-end circuit into a digital signal. Since ADCs are widely used in many different fields, their types are very diverse, and their characteristics and performance ranges are also very diverse. Therefore, it is necessary to select the most suitable ADC considering the characteristics of the sensor and its application field. An ADC for a sensor that measures the change in physical characteristics does not require high sample-per-second (SPS) performance because of the characteristics of the sensor. Most sensors require a sampling rate of several tens of SPS to several kSPS, and a slightly higher (200 kSPS) is required for a microphone that measures acoustic signals. For touch sensors arranged as arrays, 100 to 10,000 sensors are placed on one panel based on the size of the display panel used. The report rate, which is the measurement rate for the entire panel, requires 60–180 Hz; therefore, the SPS required for the ADC in touch applications is several kSPS to several MSPS.

The resolution of the ADC is expressed by the number of bits of the ADC. Because the Signal-to-quantization-noise ratio(SQNR) that can be achieved with a specific number of bits has the value

(11) SQNR=6.02·bits+1.76 dB

The number of bits should be determined such that they have a higher SQNR performance than the SNR performance of the analog front-end circuit.

In general, the sensor and analog front-end circuit have an SNR of 60–120 dB. In the case of a touch sensor, there is a considerable amount of noise that comes from the outside, and therefore, it has an SNR of 30–80 dB. Equation (12) must be satisfied for the ADC to not become the bottleneck of the overall readout system performance.

(12) $$\mathrm{Bits}>\frac{\mathrm{SNR}-1.76\;\mathrm{dB}}{6.02}$$

A resolution of 10–20 bits is required to satisfy the above SNR range; 5–13 bits are required for a touch sensor.

**Figure 8:**
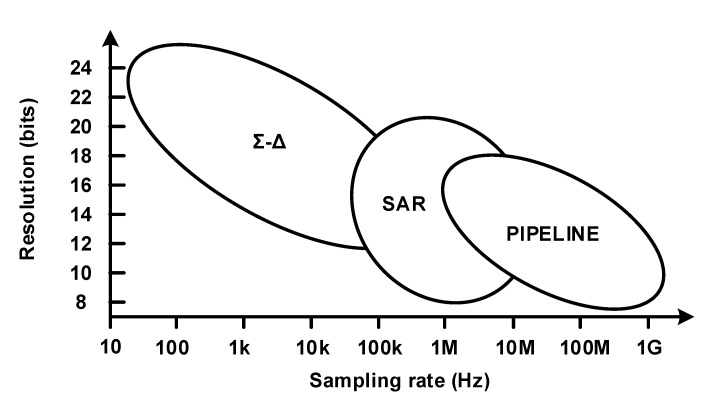
Analog-to-digital converter(ADC) architectures, resolution, and sampling rates [8].

Figure 8 shows the approximate sampling rate and resolution characteristics of three types of ADCs: pipeline, SAR, and delta-sigma, which are commonly used as ADC structures. A delta-sigma ADC is used when tens of SPS to several kSPS sampling rates with 16 bits or more resolution are required; when several tens of kSPS sampling rates with 16 bits or less resolution are required or when power consumption is important, SAR ADC is used.

In the case of the delta-sigma ADC, a high SNR is achieved through a noise-shaping effect and oversampling. SQNR is the theoretical SNR only considering quantization noise, and it is affected by the number of bits of the comparator and DAC(N), the delta-sigma loop order(L), and the oversampling ratio(OSR). SQNR is calculated as

(13) $$\mathrm{SQNR}\;[\mathrm{dB}]=6.02\cdot N+1.76+\left(20L+10\right)\log_{10}\mathrm{OSR}-10\log_{10}\left(\frac{\pi^{2L}}{2L+1}\right)$$

The resolution, loop order, and OSR of the delta-sigma ADC were determined based on the target SNR. The delta-sigma ADC can achieve a very high SQNR depending on its configuration; however, there are disadvantages in that the power consumption increases because the sampling frequency increases in proportion to the oversampling ratio, and an amplifier with a high bandwidth is required.

The SAR ADC has an advantage in that it is easy to implement low power compared to other ADCs by using a capacitor DAC. The SAR ADC performs a binary search from most significant bit (MSB) to least significant bit (LSB) with the charge-sharing operation of the capacitor DAC, and it then performs the analog-to-digital conversion. Because the capacitor DAC does not require an amplifier, it is advantageous in terms of power consumption. However, the SAR ADC does not apply noise shaping such as the delta-sigma ADC, and therefore, the SQNR increase caused by oversampling is small compared to the delta-sigma ADC, as shown by

(14) $$\mathrm{SQNR}[\mathrm{dB}]=6.02\cdot N+1.76+10\cdot \log_{10}\mathrm{OSR}$$

That is, when an ADC of the same resolution is used, the SQNR is lower than that of a delta-sigma ADC.

### 2.4. Post-Processing

The sensor readout system uses a post-processor to achieve higher performance or support various additional functions by removing noise and errors difficult to remove using only an analog front-end circuit through a digital-domain operation. Errors that are difficult to remove using the analog front-end circuit alone include external noise in the frequency within the sensing signal band, errors caused by sensor mismatches, and errors of the ADC. It is not only difficult to design a circuit to cope with these errors, but it is also disadvantageous in terms of power consumption and area as the analog filter is vulnerable to nonlinearity. However, digital-domain filters are relatively less affected by these problems [9]. Further, post-processing has the advantage of improving performance even after chip manufacturing by enabling programmable changes to the filter according to the environment or user needs through a firmware update.

Post-processing allows compensating sensor characteristics such as the temperature variation of the sensor and process variation between sensors and circuit limitations such as offset and gain error in analog front-end circuits or ADCs. Further, it compensates for external noise. In the case of touch sensors, capacitance changes may occur because of water droplets or palms, and problems that cannot be solved with capacitor sensors or analog front-end circuits alone are eliminated in post-processing by comprehensively evaluating the capacitance change pattern, intensity, and so on. Post-processing also allows calculating the touched position coordinates based on the capacitance measured by the touch sensor.

## 3. Technical Issues of the Capacitive Sensor Readout System

This section introduces the technical issues of the capacitive sensor readout system and also the solutions that have been presented for the major issues. Table 1 summarizes the technical issues for each application covered in capacitive sensor readout system studies published in major journals and conferences in the field of sensors and circuits for the past 10 years (2011–2021). The gyroscope was included in the accelerometer because of the similarity of its operation principle to that of the accelerometer; the microphone was included in the pressure sensor because it senses sound signals through pressure. Sensors that have common features for sensing the concentration of substances, such as particle meters and chemical sensors, are classified as concentration sensors.

Table 1 indicates that the SNR is a very important performance indicator for the accurate measurement of the physical quantity because the core function of the sensor readout system is to measure the physical quantity. Further, power consumption is an important consideration because capacitive sensors are used extensively in small devices. Sensing speed is not a major design issue because the rate of change of the physical quantity to be measured is not fast in most sensor systems. However, touch applications operate as a matrix based on the time-division method. The number of channels and parasitic R and C increase as the physical size of the sensor increases; further, the report rate requirement for smooth touch increases. Thus, unlike in other sensor readout systems, sensing speed issues should be considered. However, the sensitivity is related to the performance of the sensor itself rather than the readout system.

### 3.1. Signal-to-Noise Ratio (SNR): Signal Power

**Figure 9:**
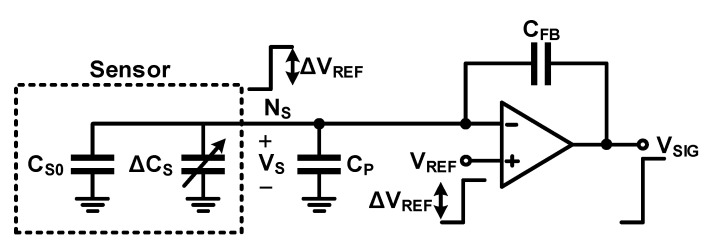
Base and parasitic capacitances in a self-capacitance readout circuit.

It is necessary to increase the signal power or reduce the noise power to achieve a higher SNR because SNR is the ratio of signal power to noise power. Conversely, SNR is lowered when the signal power is degraded or noise is increased. Figure 9 shows the capacitive sensor and basic self-capacitance readout circuit previously introduced in Figure 5. Figure 9 shows the base capacitance (*C_S_*_0_) and capacitance change (Δ*C_S_*) caused by a physical quantity, and the parasitic capacitance (*C_P_*) between the sensor and input of the readout circuit, including the input capacitance of the readout circuit, for a capacitive sensor. When *V_REF_* is changed by Δ*V_S_*, the amount of charge on the sensor capacitor changes; the resulting Δ*Q_SIG_* is given by

(15) $$\Delta Q_{SIG}=\left(C_{P}+CS_{0}+\Delta C_{S}\right)\Delta V_{S}$$

Further, the output voltage change (Δ*V_SIG_*) caused by Δ*Q_SIG_* can be obtained as:

(16) $$\begin{array}{ll} \Delta V_{SIG} & =\frac{\Delta Q_{SIG}}{C_{FB}}+\Delta V_{S}\\ & =\left(\frac{C_{P}+CS_{0}+\Delta C_{S}}{C_{FB}}+1\right)\Delta V_{S}\\ & =\left(\frac{C_{P}+CS_{0}}{C_{FB}}+1\right)\Delta V_{S}+\frac{\Delta C_{S}}{C_{FB}}\Delta V_{S} \end{array}$$

Equation (16) indicates that Δ*V_SIG_* includes not only the terms caused by sensor capacitance change (Δ*C_S_*) but also the term caused by the parasitic and base capacitances. If *C_P_* and *C_S_*_0_ are large, a large feedback capacitor (*C_FB_*) must be used to maintain Δ*V_SIG_* within the power supply range without clipping. Accordingly, there is a problem in that the amount of voltage change ($\frac{\Delta C_{S}}{C_{FB}}\Delta V_{S}$) in response to the change in the capacitance of the sensor decreases, which results in a decrease in signal power.

**Figure 10:**
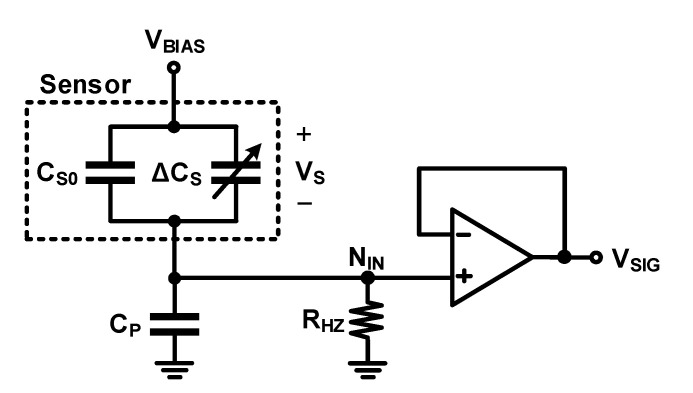
Base and parasitic capacitances in a DC-biased self-capacitance readout circuit.

A DC-biased self-capacitance readout circuit is used for sensors that require the DC bias (*V_BIAS_*), as shown in Figure 6. Figure 10 shows the sensor, *C_P_*, and readout circuit. The output of this circuit is also affected by *C_P_*.

(17) $$\Delta V_{SIGNAL}=\frac{\Delta C_{S}}{C_{S0}+C_{P}}V_{BIAS}$$

Equation (17) indicates that the signal power decreases with an increase in *C_P_* and *C_S_*_0_. It is not possible to reduce *C_S_*_0_ in the analog front-end circuit because it is determined by the sensor design. However, *C_P_* is affected by the physical design, such as the routing metal layout of the connection between the sensor and the circuit, because *C_P_* occurs between the sensor output and the input of the readout circuit along with the input capacitance of the readout circuit. When designing the layout connecting the sensor and readout circuit, efforts should be made to minimize the parasitic capacitance. Despite these efforts, the gate capacitance of input transistor, the parasitic capacitance of the pad, and routing metal remain.

#### 3.1.1. Mutual-Capacitance Sensing

**Figure 11:**
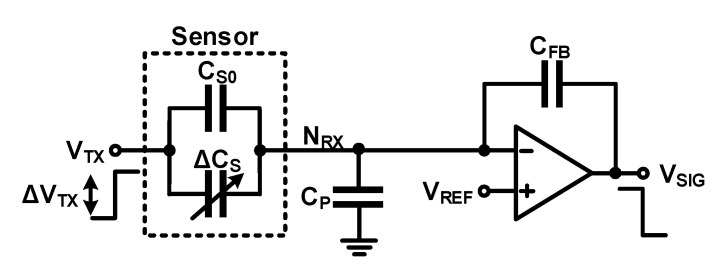
Base and parasitic capacitances in a mutual-capacitance readout circuit.

A mutual-capacitance sensing method is widely used for many sensors to solve the problem of signal power reduction caused by the parasitic capacitance of self-capacitance sensing. In the mutual-capacitance sensing method, the TX node (*N_TX_*) that applies voltage to the sensor capacitor and the RX node (*N_RX_*) that measures the change in charge are separated. As shown in Figure 11, the voltage at node *N_RX_* is fixed to *V_REF_* by the Op Amp. When a driving voltage (Δ*V_TX_*) is applied to the sensor, the voltage across the sensor capacitor and the amount of charge changes; however, the voltage does not change at the parasitic capacitor in *N_RX_*, and the parasitic capacitor does not affect the change in the amount of charge or the output signal (Δ*V_SIG_*), which is expressed as Equation (7). However, the effect of parasitic capacitance on mutual capacitance sensing is not fully eliminated. *V_RX_* is not exactly equal to *V_REF_* and varies depending on the output voltage of Op Amp (*V_SIG_*) because the real Op Amp does not have an infinite open-loop gain (*A*).

(18) $$\Delta V_{SIG}=-\frac{A}{A+1}\frac{\left(C_{S0}+\Delta C_{S}\right)}{\frac{C_{S0}+C_{P}}{A+1}+C_{FB}}\Delta V_{TX}$$

The Δ*V_SIG_* includes the open-loop gain of Op Amp, shown in Equation (18). The equation indicates that the influence of *C_P_* is almost reduced by the open-loop gain; however, it does not completely disappear.

For sensors that require the DC bias used in some applications, it is difficult to use a mutual-capacitance readout circuit because a large signal cannot be applied to the TX node. Further, the effect of *C_P_* was eliminated, but the effect of *C_S_*_0_ was not.

#### 3.1.2. Charge-Balancing Compensation: Using a Compensation Capacitor

**Figure 12:**
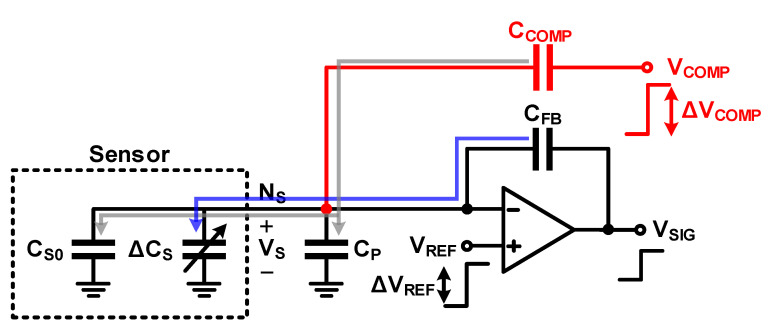
Charge-balanced compensation using a compensation capacitor in a self-capacitance readout circuit.

A charge-balancing compensation technique using a compensation capacitor (*C_COMP_*) can be used to eliminate the effect of *C_S_*_0_. Figure 12 shows the charge-balancing compensation circuit using *C_COMP_* in the self-capacitance readout circuit. In this circuit, *C_COMP_* is added to the node where the parasitic capacitance occurs (*N_S_*), and the voltage on the other side of *C_COMP_* (*V_COMP_*) is driven in the same phase and polarity as the voltage of node *N_S_*. If the change in charge amount changes because of *C_S_*_0_ and *C_P_* (Δ*Q_OFF_*) and the change in charge amount caused by Δ*V_COMP_* and *C_COMP_* (Δ*Q_COMP_*) are determined to be the same, Δ*Q_OFF_* is supplied by *C_COMP_* and not the feedback capacitor (*C_FB_*). This is formulated as

(19) $$\begin{array}{ll} \Delta Q_{OFF} & =\left(C_{P}+C_{S0}\right)\Delta V_{S}\\ & =\Delta Q_{COMP}\\ & =C_{COMP}\left(\Delta V_{COMP}-\Delta V_{S}\right) \end{array}$$

The voltage of Δ*V_COMP_* required for this can be obtained as

(20) $$\Delta V_{COMP}=\left(\frac{C_{P}+C_{S0}}{C_{COMP}}+1\right)\Delta V_{S}$$

Further, the influence of *C_S_*_0_ and *C_P_* on the change in output voltage (Δ*V_SIG_*) is removed by the charge-balancing compensation, and only the change in sensor capacitance is reflected.

(21) $$\begin{array}{ll} \Delta V_{SIG} & =\frac{\left(C_{S0}+C_{P}\right)\Delta V_{S}-C_{COMP}\left(\Delta V_{COMP}-\Delta V_{S}\right)}{C_{FB}}+\frac{\Delta C_{S}}{C_{FB}}\Delta V_{S}+\Delta V_{S}\\ & =\left(\frac{\Delta C_{S}}{C_{FB}}+1\right)\Delta V_{S} \end{array}$$

**Figure 13:**
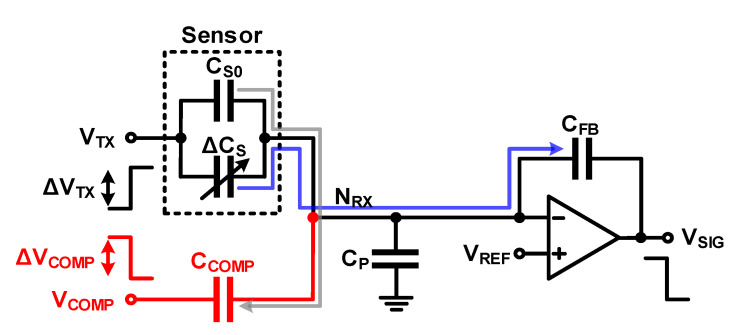
Charge-balanced compensation using a compensation capacitor in a mutual-capacitance readout circuit.

A charge-balancing compensation method can be applied to eliminate the effect of the base capacitance in both self-capacitance sensing and mutual-capacitance sensing (Figure 13). When applied to a mutual capacitance readout circuit, the effect of the base capacitance can be removed if the sum of the charge due to the base capacitance (*C_S_*_0_Δ*V_TX_*) and the charge due to the compensation capacitor (*C_COMP_*Δ*V_COMP_*) is 0, as

(22) $$\begin{array}{ll} \Delta V_{SIG} & =-\frac{C_{S0}+\Delta C_{S}}{C_{FB}}\Delta V_{TX}-\frac{C_{COMP}}{C_{FB}}\Delta V_{COMP}\\ & =-\frac{C_{S0}\Delta V_{TX}+C_{COMP}\Delta V_{COMP}}{C_{FB}}-\frac{\Delta C_{S}}{C_{FB}}\Delta V_{TX}\\ & =-\frac{\Delta C_{S}}{C_{FB}}\Delta V_{TX}\;\;\;,when\;\;\Delta V_{COMP}=-\frac{C_{S0}}{C_{COMP}}\Delta V_{TX} \end{array}$$

When using the method with the additional *C_COMP_*, the compensation charge amount and the charge amount by *C_S_*_0_ and *C_P_* must exactly match to be removed. Therefore, for accurate compensation, either *C_COMP_* is implemented as a variable capacitor or *V_COMP_* is implemented as a DAC [10]. Further, SAR logic is used to determine the correct compensation charge [11,12]. Even in the case of variable capacitors and DACs, there is a limit in that the perfect compensation is difficult because of the limitation of the resolution. In addition, when using signals of other waveforms such as sine waves instead of accurate pulses such as Δ*V_REF_* in the self-capacitance readout circuit or Δ*V_TX_* in the mutual-capacitance readout circuit, there may be an error source if the accurate negative polarity of compensation voltage driving is not realized. To solve this problem, a structure that compensates for a negative capacitance circuit using an additional Op amplifier without applying a negative polarity signal has been proposed [13,14,15].

#### 3.1.3. Charge-Balancing Compensation: Using a Differential Sensor

**Figure 14:**
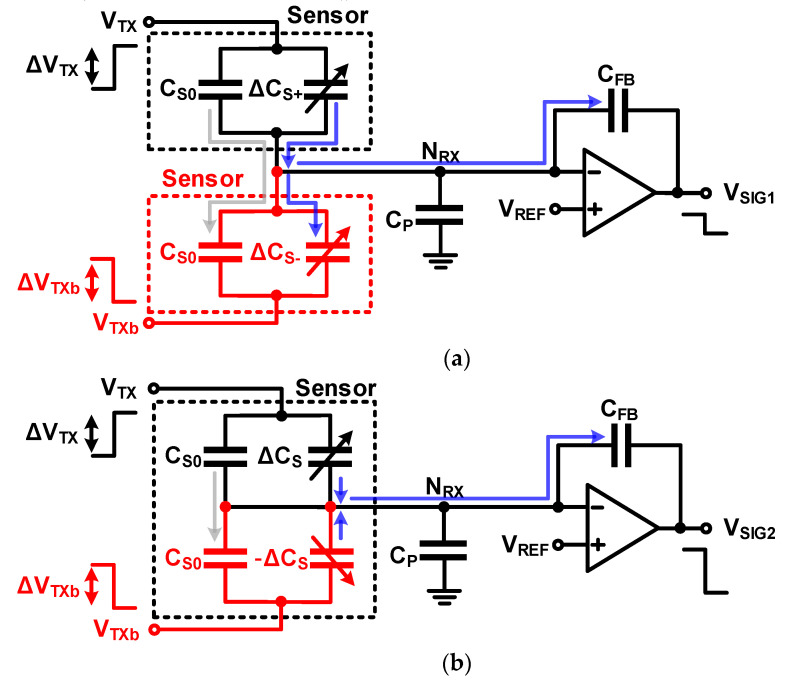
Charge-balanced compensation using a compensation capacitor in a mutual-capacitance readout circuit. (**a**) configured identical sensors such that the base capacitance; (**b**) a single sensor composed of two differential capacitors.

Another method of compensation uses an another sensor instead of a compensation capacitor. Figure 14 shows such a charge-balancing circuit. There exists a method to configure two identical sensors such that the base capacitance is the same as that shown in Figure 14a or a single sensor composed of two differential capacitors, as shown in Figure 14b. Instead of balancing the charge from the added compensation capacitor and the charge from the base capacitance in the sensor, the base capacitance of the two sensors are very similar even if they are not completely matched because both methods in Figure 14 use the same two sensors; thus, an improved compensation is possible. The output signal (Δ*V_SIG_*_1_) when two identical sensors and output signal (Δ*V_SIG_*_2_) when a differential sensor are used are, respectively, given by

(23) $$\begin{array}{ll} \Delta V_{SIG1} & =-\frac{C_{S0}+\Delta C_{S+}}{C_{FB}}\Delta V_{TX}-\frac{C_{S0}+\Delta C_{S-}}{C_{FB}}\left(-\Delta V_{TX}\right)\\ & =-\frac{\Delta C_{S+}-\Delta C_{S-}}{C_{FB}}\Delta V_{TX} \end{array}$$

(24) $$\begin{array}{ll} \Delta V_{SIG2} & =-\frac{C_{S0}+\Delta C_{S}}{C_{FB}}\Delta V_{TX}-\frac{C_{S0}-\left(-\Delta C_{S}\right)}{C_{FB}}\left(-\Delta V_{TX}\right)\\ & =-\frac{2\Delta C_{S}}{C_{FB}}\Delta V_{TX} \end{array}$$

As shown in Equation (23), the difference in the capacitance change (Δ*C_S+_* − Δ*C_S−_*) between the two sensors is measured, and therefore, it is difficult to measure if a similar change in the physical quantity is applied to the two sensors. Thus, there is a limitation in that one sensor must be placed in a place that is not affected by the physical quantity or placed far away so that similar physical quantity changes do not occur in the two sensors or require post-processing. When a differential sensor is used, the difference between the two capacitances becomes rather large because the capacitance of the sensor for the same physical quantity forms a differential and reacts with opposite polarity. Therefore, in the case of the differential, it is possible to obtain the effect of increasing the signal power and removing the effect of the base capacitance.

**Figure 15:**
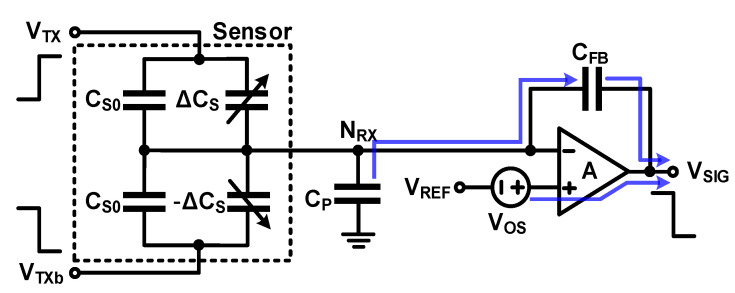
Non-idealities of Op Amp in a charge-balanced compensation circuit.

The compensation method using the sensor described above assumes that the base capacitance of the two sensors is the same; however, the effect of the base capacitance is not fully eliminated because a mismatch occurs because of process variation. Sensors and compensation capacitors are sometimes used together for charge-balancing compensation to eliminate these residual errors [16,17,18]. Further, non-ideality such as the offset and open-loop gain error of Op Amp and sensor mismatch cause the parasitic and base capacitances of the sensor to not be fully eliminated. To explain this, the offset voltage (*V_OS_*) and open-loop gain (*A*) were assumed for Op Amp, as shown in Figure 15. Even if it is assumed that the parasitic and base capacitances of the two sensors match, the output voltage error (Δ*V*_ERROR_) caused by *V_OS_* and *A* are given by

(25) $$\begin{array}{ll} \Delta V_{ERROR} & =V_{OS}+\frac{\Delta Q_{ERROR}}{C_{FB}}\\ & =V_{OS}+\frac{\left(-\frac{\Delta V_{SIG}}{A}\right)\left(C_{P}+2C_{S0}\right)}{C_{FB}} \end{array}$$

Further, Δ*V_ERROR_* can be calculated as the capacitance measurement error of the sensor as

(26) $$\begin{array}{ll} \Delta C_{S,ERROR} & =-\frac{\Delta V_{ERROR}}{\Delta V_{TX}}C_{FB}\\ & =-\frac{V_{OS}}{\Delta V_{TX}}\frac{C_{FB}}{1+\frac{C_{P}+2C_{S0}}{AC_{FB}}} \end{array}$$

Therefore, it is necessary to increase the open-loop gain (*A*) of the Op Amp and design to have a small offset to minimize the error caused by the non-ideality of the Op Amp. However, this circuit design increases the power consumption and area, which should be considered together. Oversampling successive approximation circuit structures and methods have been proposed to remove gain error using methods besides the design of a high-performance Op Amp [19,20]. These methods reduce the gain error gradually by accumulating and repeating the phase for storing and compensating the gain error several times to obtain one output result.

### 3.2. Signal-to-Noise Ratio (SNR): Internal Noise

**Figure 16:**
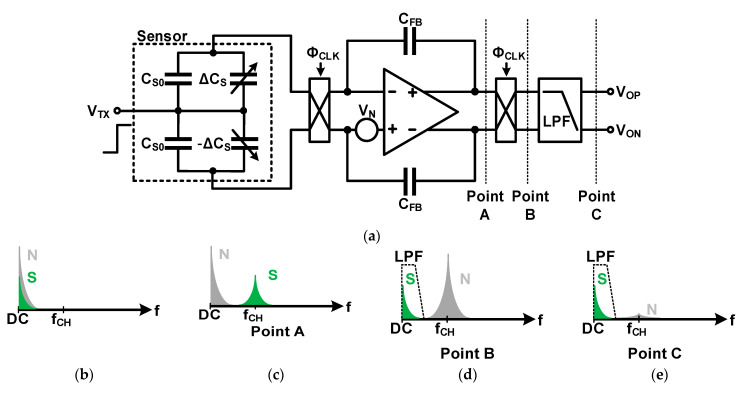
Chopper-stabilized fully differential amplifier with a differential sensor: (**a**) Circuit diagram; (**b**) signal and noise frequency without chopper stabilization; (**c**) with chopper stabilization at point A; (**d**) point B; (**e**) point C.

The internal noise of the readout system can be divided into the sensor, analog front-end circuit, and ADC noise. In the case of the analog front-end circuit, the Op Amp is the main noise source, and flicker noise is dominant because the signal frequency band is low. Chopper stabilization is widely used as a circuit technique to remove such flicker noise. Figure 16 shows an example of a circuit that applies chopper stabilization. As shown in Figure 16b, when low-frequency noise such as flicker is combined with the signal and is difficult to distinguish, as shown in Figure 16c, the signal frequency is modulated by the chopping frequency to distinguish the signal from noise. Further, as shown in Figure 16d,e, the signal that has passed the demodulation is moved to the low-frequency band, and the noise in the low-frequency band is moved to the chopping frequency. Subsequently, a low-pass filter (LPF) is applied, and only noise is removed.

Correlated double sampling (CDS) is a widely used low-frequency noise-reduction method. The CDS takes only the readout signal, which is the difference after sensing the output by the low-frequency noise and output, including both the low-frequency noise and readout signal. This method is performed in post-processing after sampling and conversion twice in ADC, or it is implemented to subtract noise in the analog circuit when measuring the readout signal after sampling the noise value into the capacitor. Alternatively, CDS can be implemented as a time-domain differential that measures each output with opposite sensor-driving polarity and then takes the difference between the two [21]. The CDS method is only applicable to low-frequency noise because the time for sampling only noise and the time for sampling a signal containing noise are not the same.

The process-voltage-temperature(PVT) variation of the sensor and analog circuit has a significant influence on the accuracy and reliability of the sensor readout system. The charge-balancing compensation method can be said to be a countermeasure against process variation through circuit design. In addition to the method of circuit design, the method of storing the output value caused by the process variation of the sensor in advance and reflecting it in post-processing is often used [22]. Temperature variation affects the capacitance of the capacitive sensor and affects the characteristics of the devices used in the readout circuit, which results in changes in the offset and gain of the Op Amp and ADC. The most common method used to eliminate the effect of temperature is to include a temperature sensor in the readout circuit to compensate for the effect of temperature change based on the measured temperature. For compensation, it is necessary to analyze how the capacitive sensor and readout system are affected by temperature [23,24]. The charge-balancing compensation method discussed in Section 3.1.2 can be used as a compensation method. The effect of temperature variation on the output voltage can be eliminated by using a compensation capacitor as a temperature sensor [25]. An orthogonal pseudo-random signal is placed in the signal path while operating, and only the output by the pseudo-random signal is separated using the orthogonal characteristic from the combined output to compensate for the gain variation of the Op Amp caused by temperature without using an additional temperature sensor. Then, the gain of Op Amp can be calculated and compensated from the separated signal [26].

### 3.3. Signal-to-Noise Ratio (SNR): External Noise

The effect of external noise on the capacitive sensor readout system is a topic addressed in touch sensors because, unlike other sensors, the noise originating from the outside is larger than the noise of the readout circuit itself in touch applications. Further, in the case of touch sensors, the area constituting the mutual capacitance is large, and the parasitic capacitance that occurs between them is very large (up to hundreds of pF) because the distance between the sensor electrode and display panel is very close and the physical size of the display/touch panel is large. Further, the pixels of TFT-LCD or AMOLED are driven with a relatively large data voltage, and therefore, the noise flowing into the touch sensor electrode is large. In addition, noise inflow is caused by the reference potential difference, such as charger noise and human noise, because the size of the sensor itself is larger than that of other sensors; touch sensors are subject to external inflow signals as noise such as an antenna. Therefore, in touch applications, the focus is on removing these external noises rather than the noise of the circuit.

#### 3.3.1. Fully Differential Structure and Chopper Stabilization

**Figure 17:**
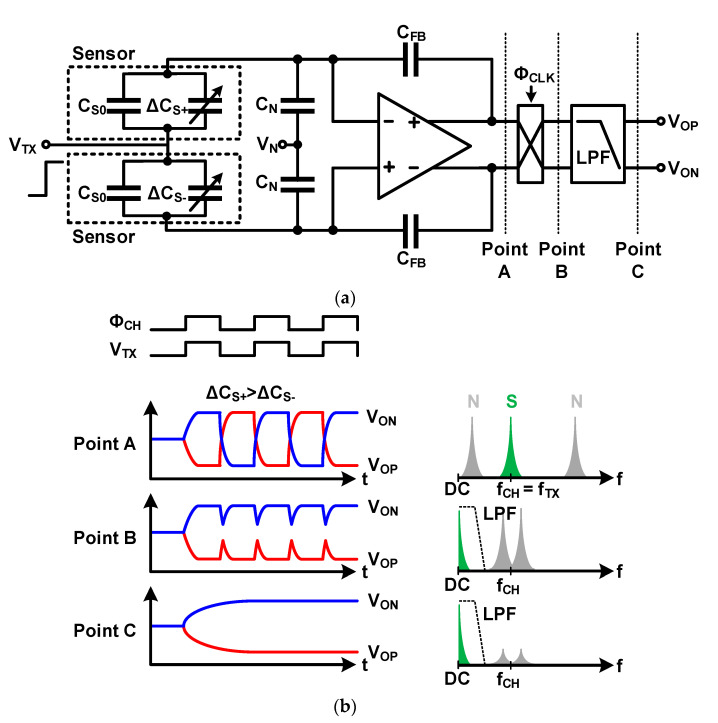
Chopper-stabilized fully differential amplifier for a touch sensor: (**a**) circuit diagram; (**b**) time-domain and frequency-domain signals.

The fully differential structure and chopper stabilization were used to remove the noise of the readout circuit, as indicated in Section 3.2. This method is also used to eliminate external noise in touch applications. Among external noises, the display noise, which has a major influence on the SNR of the touch readout system, is common noise (locally independent), and therefore, it is removed by the common-mode rejection performance of the fully differential Op Amp. The display noise entering the touch sensor can be suppressed by using two adjacent sensors in a differential structure. Although the two sensors are adjacent to each other, there is a difference in the location because of the difference in the connection of the signal lines, and the incoming noise is slightly different. Chopper stabilization used to remove external noise can be implemented using the circuit shown in Figure 17a. As shown in the operation example in Figure 17, the signal from the sensor is demodulated to DC using the chopping frequency (*f_CH_*), which is the same as the frequency of the sensor-driving signal (*f_TX_*); only the sensor signal in the low-frequency band is left using an LPF. The modulated noise with the high-frequency band is removed by the LPF. Thus, not only the external noise but also the noise of the readout circuit, such as offset error, are removed. Recent studies used bandpass filters (BPFs) before demodulation and LPF to achieve higher noise-reduction performances [27,28]. This method can remove noises at different frequencies from signals; however, there is a limit in that it is difficult to remove noises that exist over the signal frequency. To solve this problem, instead of a fixed chopping frequency, a method to find and change the optimal chopping frequency to remove noise, and a method for applying pseudo-noise to the chopping frequency to provide a dithering effect to the chopping frequency, were proposed [29,30].

#### 3.3.2. Averaging, Integration, and Parallel Driving Method

**Figure 18:**
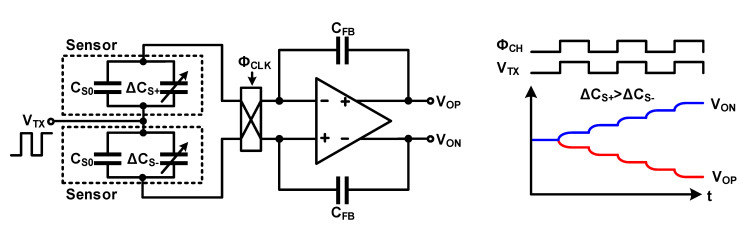
Fully differential amplifier with integration in a mutual-capacitance readout circuit.

Random noise has white noise characteristics evenly distributed over the entire frequency range. It is not easy to remove noise through a frequency filter because noise is mixed at the same frequency as the signal. In this case, the effect of noise with the same frequency as the signal can be reduced through integration and averaging. If a sensor is sensed multiple times instead of only once, a constant value is measured for the signal because of the change in the amount of the sensor; however, the random noise can ideally be eliminated by averaging the repeatedly measured output values. Accumulating the results of repeated sensing through the configuration shown in Figure 18 also has the same average effect on random noise. Further, there is a method to remove the periodic display noise with an average effect by using periodicity inversely [31]. Both the method of the repeated measurement for averaging the output value and the method of integration have a disadvantage in that the sensing time is increased by the number of repetitions compared to the one-time sensing method. Further, the random noise removal performance shows higher performance as the number of repetitions increases; however, there is a problem in that it is difficult to increase the number of repetitions because the touch sensor is composed of an array, and there are many sensors that need to be measured.

**Figure 19:**
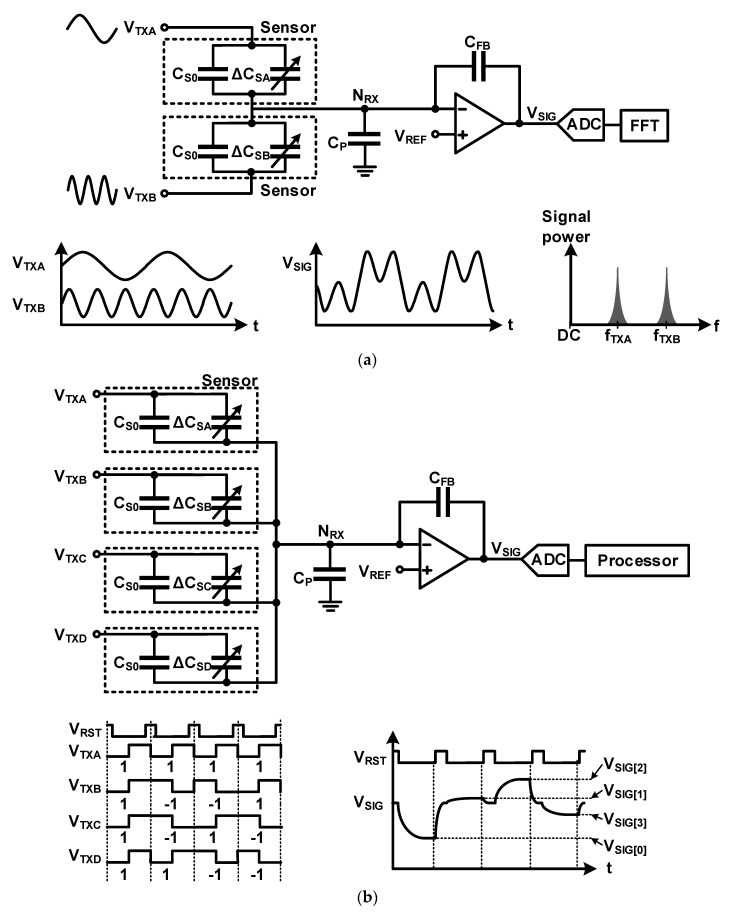
Parallel driving method: (**a**) multiple-frequency driving; (**b**) code-division multiple sensing.

A parallel driving method (PDM) that simultaneously senses multiple sensors in each channel has been developed to overcome this time constraint problem. Sensing the sensors of each channel with time division is an existing general method that is similar to time division multiple access (TDMA) used in communication. Unlike the general method, PDM is similar to code division multiple access (CDMA) and frequency division multiple access (FDMA) used in communication. For multiple-frequency driving similar to FDMA, as shown in Figure 19a, signals with different frequencies are applied to several TX channels simultaneously in parallel, and the combined signals of various frequencies are measured from the readout circuit of each channel; further, each sensor is measured simultaneously by separating it by frequency through post-processing such as fast Fourier transform (FFT) [32]. Orthogonal frequency division multiplexing (OFDM) can be applied because the frequencies used for driving may interfere with each other [33]. For code-division multiple sensing similar to CDMA, orthogonal signals are applied to each TX line, and the output value for each sensor is separated through post-processing calculations [34,35]. Figure 19b shows an example using the 4 × 4 matrix Walsh–Hadamard matrix. In Figure 19, the values from all four sensors are superimposed on the output; however, because the applied code has an orthogonal characteristic, it can be separated by calculation. In the example, the code is expressed as a pulse; a continuous-time AC signal can be encoded as a code and used instead [36]. Because all PDM methods measure multiple sensors in the array simultaneously, the average readout time does not increase. However, depending on the code, the output signal may be saturated to output range. To prevent this from occurring, efficient orthogonal code was proposed [27,37,38,39,40,41]. As an additional problem, if the same code is repeatedly used for PDM, energy is concentrated at a specific frequency, and therefore, it may not be able to remove random noise along with the EMI problem properly. A method for creating and using a pseudo-random orthogonal code generator was proposed to solve this problem [42]. The disadvantages of these PDM methods are that the area and power consumption of a sensor-driver circuit required for creating and applying multiple frequencies and a digital circuit for encoding and decoding orthogonal codes increase. Depending on the code, the output signal may become saturated beyond the output range as the signals for several sensors overlap. A more efficient orthogonal code was proposed [27,37,38,39,40,41]. PDM method may not be able to properly remove random noise along with the EMI problem if the same code is repeatedly used and concentrated at a specific frequency. To solve this problem, a method of creating and using a pseudo-random orthogonal code generator has been proposed [42]. The disadvantages of these PDM methods are that the area and power consumption of a sensor-driver circuit for creating and applying multiple-frequency and a digital circuit for encoding and decoding orthogonal codes increase.

#### 3.3.3. Frequency Adaption

**Figure 20:**
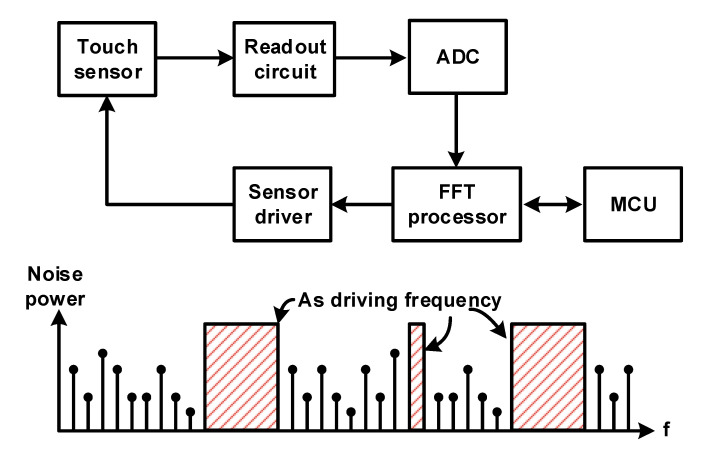
Concept of frequency adaption technique for capacitive sensor readout system.

Although various driving methods and circuit techniques have been introduced to cope with external noise, there is an underlying limitation that noise cannot be properly removed when the signal and noise frequencies overlap. It is difficult to expect a noise reduction effect by averaging and integrating non-random noise. It may be possible to determine a noise-free frequency band and use it as the sensor-driving frequency if the noise characteristics of the display panel and environment where the touch sensor will be used can be identified in advance. In touch applications, a large amount of external noise is introduced because of the large size of the display panel, and in the case of mobile devices, the size and frequency of the incoming noise vary greatly depending on the location where it is used. Therefore, using a specific sensor-driving frequency and reducing noise is not a realistic solution. As shown in the example spectrum of Figure 20, frequency adaptation is a method wherein the readout system identifies and uses a frequency that is good for driving and sensing the sensor because it has the least noise among various frequency bands. As shown in the block diagram of Figure 20, the readout system measures the noise and uses the FFT processor and MCU to determine the frequency band with the least noise, which decides to use the frequency band as the sensor-driving frequency [30,32]. Through this operation, optimal SNR performances can be achieved even in various noise environments.

#### 3.3.4. Post-Processing

The touch application not only senses the capacitance change but also determines whether a touch is made by the user’s finger. Besides human fingers, various physical noise sources, such as water or palms, can come into contact with the touch panel. These sources have a certain amount of electric charge similar to that for a finger, and this can affect the capacitance of the touch sensor. It is very difficult to detect and remove those physical noise sources from the analog front-end circuit, and therefore, the role of post-processing is important in touch applications because it can determine whether it is a finger touch or not by comprehensively evaluating the amount of capacitance change and the changed distribution pattern in the array [43,44]. Post-processing is an operation performed in the digital domain, and therefore, if an improved noise removal algorithm is developed, it has the advantage in that it can be used in an existing system via a firmware update.

### 3.4. Power Consumption

Power consumption is an important issue in addition to the SNR in the capacitive sensor readout circuit. The importance of mobile and IoT devices cannot be overemphasized. In a readout system for capacitive sensors, power consumption can be divided into sensor drivers, analog front-end circuits, ADC, and logic circuits for post-processing.

The power of the sensor driver is determined by the capacitance of the sensor, excitation voltage, and driving frequency. A low excitation voltage can be used to lower the power consumption; however, there is a problem in that the signal power can also be reduced. The method of reducing power consumption by lowering the sensor-driving frequency can be used as a method to significantly reduce power consumption in touch applications where the parasitic capacitance is very large because it is composed of a large-area array [45]; further, a method of reducing driving energy through stepwise driving can also be used [46]. For analog front-end circuits, there is a limit to lowering power consumption because of the trade-off between power consumption and noise. If the method of increasing the signal power discussed in Section 3.1 is applied, there is room for reducing power consumption because the noise performance required to achieve the same SNR is alleviated [47]. Alternatively, a structure that removes the power of the pre-amplifier with a capacitance-to-digital (CDC) that connects the ADC directly to the sensor without using the pre-amplifier or a structure that eliminates overlapping circuits by combining the analog front-end pre-amplifier and ADC is suggested to reduce the power consumption of analog front-end circuits [48,49,50,51,52].

When adopting ADC for capacitive sensor readout systems, SAR ADCs with basic low-power characteristics are the most advantageous in terms of power consumption. After sampling the input, the SAR ADC performs a binary search and compares the number of binary bits required for each step by charge sharing through the capacitor DAC. For this, the sensor undergoes the charge–discharge cycle for every sample, and charge sharing of the capacitor DAC occurs at every conversion step. Therefore, a method to reduce the conversion energy required per sample by reducing the conversion step or the number of samplings is used [53,54].

The delta-sigma ADC used when a higher SNR is required employs a higher oversampling ratio for a higher noise shaping effect; therefore, it has a disadvantage in that it consumes significant power as the sampling frequency is increased. Therefore, it is very important to reduce the power consumption of the delta-sigma ADC in the readout circuit. The bandwidth of the Op Amp must be increased to drive at a high driving frequency, which leads to an increase in the power consumption of the Op Amp. Therefore, a structure that does not need to increase the bandwidth of the Op Amp is adopted to lower the power consumption of the delta-sigma ADC [55], or an inverter-based amplifier is used to lower the power consumption and maintain a high bandwidth [56].

**Figure 21:**
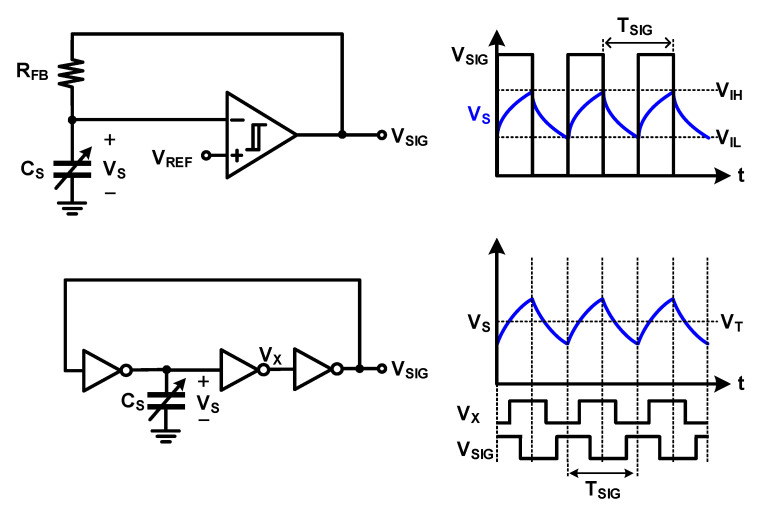
Operation of voltage-controlled oscillator.

Recently, considerable research has been conducted on the method for lowering the supply voltage to lower the power consumption in other fields. In the field of capacitive sensor readout, analog circuits that represent information in the time domain instead of expressing information as a voltage amplitude and operate at a low supply voltage have also been proposed [57,58,59,60]. As one of these methods, an oscillator-based voltage-controlled oscillator (VCO), as shown in Figure 21, is sometimes used as an ADC instead of a traditional voltage-based ADC. The VCO can operate at a low supply voltage because it is fabricated as a current hungry inverter; further, it consumes little power and can achieve high resolution because the conversion resolution is in the time domain. However, it is difficult to apply the method of increasing the signal power introduced in Section 3.1, the method of reducing flicker noise and thermal noise, and the method of compensating for external noise introduced in Section 3.2 and Section 3.3. In addition, the linearity is poor because of the characteristics of the VCO. To overcome these shortcomings, methods such as using the PLL structure [58], applying chopper stabilization [59,60], and implementing the delta-sigma modulator with VCO [60] have been proposed.

Recently, efforts to reduce power consumption through transistor-level design such as analog front-end circuits and ADCs and methods for lowering power consumption at the algorithm level considering actual applications are studied. In the case of a microphone, the performance and power consumption are lowered when there is no sound input to the readout system, and only the input of the sound signal is detected with a low SNR. When the sound input is detected, the operation algorithm is programmed to quickly return to the normal state and sense the input sound signal with a normal SNR. Therefore, a method that achieves very low power consumption in an actual use environment is suggested [61].

## 4. Design Example

A design example of a capacitive sensor readout circuit for a MEMS microphone system will be presented in this section, where it can be found how the design techniques are applied to a real sensor readout system. There has been significant growth in the demand for microphone sensors owing to the growth of voice recognition services for various home appliances, including smartphones, and voice recognition AI speakers. Recently, cordless earphones that employ two or more microphones for voice recognition and calls have been developed, and the use of four to six microphones for anti-noise cancellation (ANC) functions is gaining popularity. Hearing aids are similar to cordless earphones, and the demand is expected to increase rapidly in the future because of the aging population. The MEMS microphone sensor, which is smaller than other microphone sensors, is used because the size of these two products is very important. Further, a low noise level and low power consumption are important requirements for voice recognition, ANC function, and battery life.

The capacitor of the capacitive MEMS microphone sensor comprises a flexible diaphragm made of a membrane and a fixed back plate. A high-voltage bias must be applied between the diaphragm and the back plate to operate the sensor by generating an appropriate electrostatic force. A bias voltage of 5–20 V is required in general, and the sensor is directly affected by the bias voltage, as shown in Equation (5). The bias voltage fluctuations change in the gain of the sound being converted to voltage, and therefore, the linearity may deteriorate. Thus, an analog front-end circuit with a high-impedance DC bias (Figure 6b) is used to apply a steady DC bias to the sensor while sensing the change in the sensor capacitance. However, in this sensing method, signal degradation is caused by parasitic capacitance, as indicated in Equation (17). Therefore, signal power degradation caused by the parasitic capacitance must be considered in the design to achieve a high SNR.

**Figure 22:**
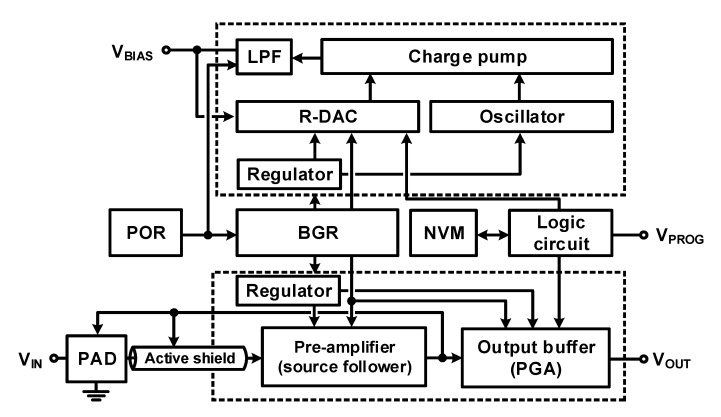
Block diagram of analog frontend for MEMS microphone.

Figure 22 shows a block diagram of the designed overall readout system. The sensor is connected to an ESD pad with a built-in back-to-back diode for high-impedance DC bias; a source follower is used as a pre-amplifier to drive the PGA, and it is used as an output buffer for proper gain and low output impedance. A charge pump is used to generate the bias voltage required for membrane capacitive sensor operation. It has an additional regulator (LDO) and is placed away to avoid mutual interference with the readout block. Nonvolatile memory (NVM) is used to program the DC bias and signal gain, and a logic circuit for NVM operation and one-wire data communication is used.

### 4.1. Design for Minimizing Signal Power Degradation

Input capacitance includes the input capacitance of the pre-amplifier and parasitic capacitances caused by the bonding wire, input pad, and routing. It is important to minimize the input capacitance of the readout system to minimize signal power degradation. However, the flicker noise of the input transistor in the pre-amplifier is the dominant noise in the analog front-end circuit because the acoustic signal has a relatively low-frequency band (10–100 kHz). It is necessary to design the area of the input transistor in the pre-amplifier as far as possible to reduce flicker noise; therefore, minimizing the other input capacitances (parasitic capacitance of the bonding wire, input pad, and routing) becomes more important.

**Figure 23:**
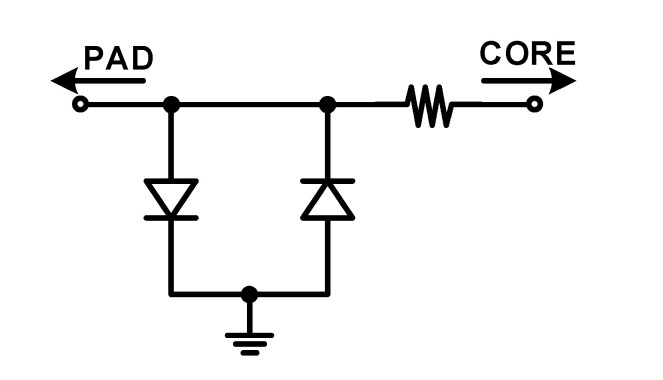
Input pad circuit using back-to-back diode.

The wire should maintain its distance from other wires to minimize the parasitic capacitance of the bonding wire. The input pad that connects the sensor and analog front-end circuit is placed away from other pads to minimize the parasitic capacitance of the bonding wire. The input pad needs to have lower parasitic capacitance and should protect the ESD from the outside. Instead of the pad IP provided by the manufacturer with the high ESD protection and a large parasitic capacitance, the following techniques were applied to the custom-designed input pad. (1) As shown in Figure 23, the back-to-back diode required for high impedance bias in the analog front-end circuit is arranged in the pad to serve the ESD diode. The size of the diode and resistor are determined to satisfy the human body model (HBM) of 500 V through simulation. (2) An active shield metal is added to minimize the parasitic capacitance in the customized input pad. (3) The routing between the pad and the pre-amplifier is made as short as possible by placing the input pad close to the pre-amplifier; further, an active shield metal is placed around the routing. (4) Dummy metal to resolve the design rules for density is not placed around the input pad and its routing.

Table 2 summarizes the post-layout simulation results on the effect of parasitic capacitance and signal degradation based on whether an active shield is applied among the customized pad and the pad IP provided by the manufacturer.

### 4.2. Design for Noise Reduction

**Figure 24:**
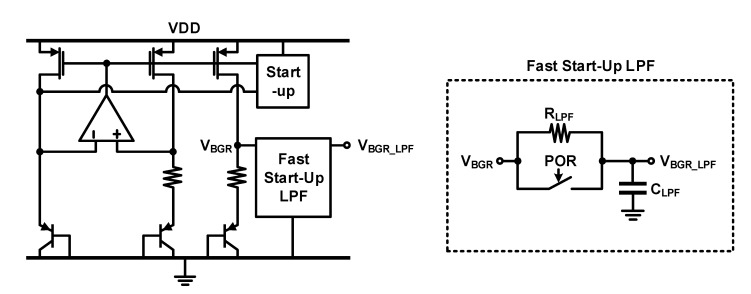
BGR circuit and fast start-up LPF.

**Figure 25:**
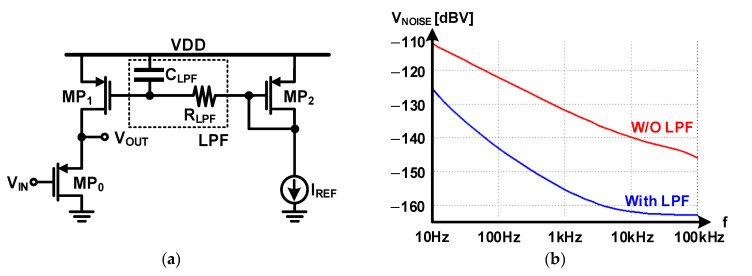
Pre-amplifier with LPF: (**a**) schematic; (**b**) noise simulation result.

As shown in the block diagram of Figure 22, the pre-amplifier and PGA are affected by the noise of the bandgap reference (BGR) through the reference voltage or reference current. A lower noise of the BGR is required to lower the noise of the entire readout circuit. Further, an LPF was added to the output of the BGR to lower the noise, as shown in Figure 24. The cut-off frequency of the LPF must be very low because the frequency band of the sound signal is low; further, a very low cut-off frequency makes the start-up settling of the BGR very slow. The power-on reset (POR) and a switch were used to settle when power was applied for overcoming the slow start-up settling, as shown in Figure 25. Using the noise simulation function of Synopsys’ hspice simulator, the noise level of the BGR for the frequency range of 10–100 kHz in the post-layout was −91.38 dBV without the LPF and −101.92 dBV with the LPF. The reduction was confirmed to be 10.54 dBV.

Flicker noise is necessary because the frequency range of the acoustic signal is very low (10–100 kHz). The flicker noise of a transistor is inversely proportional to the gate capacitance. The size of the input transistor of the pre-amplifier was designed to be as large as possible to minimize flicker noise. Further, LPF was added to the structure of the pre-amplifier to reduce the noise transmitted from the current reference, as shown in Figure 25a. The noise level of the designed pre-amplifier and the effect of the added LPF were confirmed by a post-layout noise simulation. The result of the noise simulation is shown in Figure 25b, where the noise for the 10–100 kHz frequency range is significantly reduced if LPF is present. The total noise level within the frequency range of 10 Hz–100 kHz was −95.42 dBV without LPF and −113.00 dBV with LPF, which confirmed an 18.58 noise-reduction effect.

**Figure 26:**
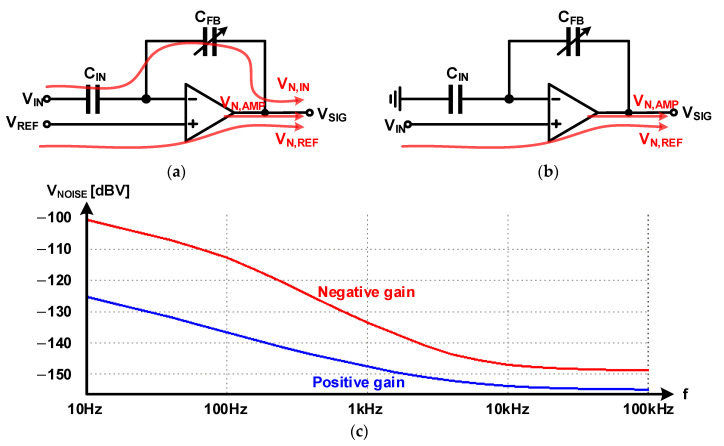
Noise in PGAs: (**a**) negative gain; (**b**) positive gain; (**c**) post-layout noise simulation result.

The output signal of the pre-amplifier is connected to the PGA, which acts as an output buffer. PGA uses a capacitive closed-loop multiplier with a negative gain, as shown in Figure 26a. A multiplier composed of an input capacitor and feedback capacitor is used to separate the output operating point of the pre-amplifier and the input operating point of the PGA. As shown in Figure 26a, the general negative gain multiplier follows the reference input; the noise from the preamplifier and the noise of the reference voltage are included in the output. As shown in Figure 26b, the positive gain multiplier does not receive the reference voltage input, and therefore, the noise of the reference voltage is not reflected in the output. The post-layout noise simulation of the negative gain and positive gain multipliers using the same Op Amp and same BGR reference are shown in Figure 26c. The total noise level for the 10 Hz–100 kHz frequency range was −85.96 dBV for the negative gain multiplier and −103.28 dBV for the positive gain multiplier; it was 17.32 dB lower than that with the positive gain multiplier.

### 4.3. Measurement

**Figure 27:**
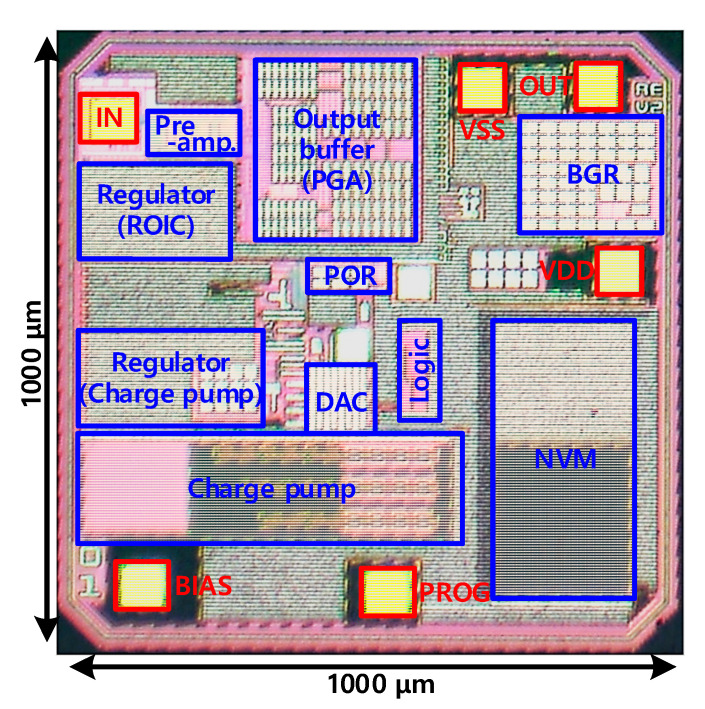
Micrograph of the fabricated MEMS microphone readout chip.

**Figure 28:**
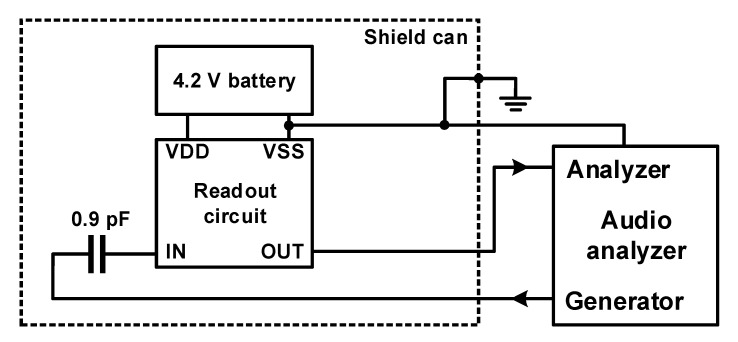
Measurement setup of the MEMS microphone readout chip.

The fabricated MEMS microphone readout circuit had a size of 1000 μm × 1000 μm, as shown in Figure 27. Figure 28 shows the environment for measuring the readout circuit. An Agilent-U8903A audio analyzer was used as the measurement equipment, and the A-weighting unit reflecting the characteristics of the audible frequency used in sound measurement was applied. Signal degradation cannot be checked when the low-impedance generator of the measuring device is directly connected to the input of the circuit because signal degradation occurs in the readout circuit owing to the parasitic capacitance of the input node. Therefore, using a discrete capacitor that corresponds to the base capacitance of 0.9 pF of the microphone sensor, an environment for AC coupling the signal of the generator was constructed. When measuring the sensitivity and SNR of the microphone, the standard for measuring the output of an acoustic signal with a size of 94 dB SPL or 1 Pa and a frequency of 1 kHz was followed [62]. Sensitivity refers to the size of the microphone signal for the sound of 94 dB SPL or 1 Pa pressure; the ratio of sensitivity and noise level becomes the SNR of the microphone. From the acoustic performance measurement result of the microphone provided by the sensor manufacturer, the magnitude of the microphone sensor output for the 1 kHz frequency and 94 dBSPL audio input was −38 dBV; therefore, the signal input from the generator was determined to be −38 dBV. Measurements were carried out using a battery in a shield box to prevent external noise because the noise level to be measured is very low. Table 3 summarizes the post-layout simulation results and measurement results when the PGA gain values were set to 0 dB and +5 dB.

## 5. Conclusions

We reviewed various state-of-the-art technologies and challenges to be addressed in the capacitive sensor readout system. The types and characteristics of capacitive sensors and the structure of the basic readout circuit were discussed; the main issues of the capacitive sensor readout system, SNR, and power consumption were analyzed; and their limits and solutions were presented. Through several performance indicators, SNR and power consumption have become more important than others in recent mobile and IoT applications. The readout circuit has a very strong influence on the SNR, and the power consumption of the entire capacitive sensor system was discussed. A circuit design technique that can increase the signal power and/or reduce noise is important to improve the SNR. Further, it is important to consider the signal power and noise in all processes from circuit architecture, transistor-level design, chip floor planning, and physical layout. It is very challenging to achieve low power while achieving a high SNR because power consumption has a trade-off relationship with noise performance. Further, research and development of low-power-consumption ADCs are very important because the ADC consumes the largest amount of power in the readout system. In addition, post-processing in digital domains has been actively conducted to contribute to high SNR, low power consumption, and various additional functions. In conclusion, to implement a high-SNR and low-consumption capacitive sensor readout system, innovation in each part from the analog frontend (AFE), ADC, post-processing, and sensors is required.

## Figures and Tables

**Table 1:** Paper counts on technical issues of capacitive sensor readout system published in major sensor conferences and journals from 2011 to 2021.

	Sensor Application					Total
Issues	Touch	Accelerometer	Pressure	Concentration	Etc.	
SNR	12	15	2	1	3	33
Power consumption	5	1	5	1	1	13
Sensing speed	4	-	2	-	-	6
Sensitivity	1	-	-	2	2	5
Etc.	2	1	-	-	2	5
Readout method	1	1	-	1	-	3
Total	25	18	9	5	8	65

**Table 2:** Post-layout simulated comparison between PAD IP and Customized PAD.

	PAD IP	Customized PAD	
		W/O Shield	With Shield
Parasitic capacitance	2.705 pF	0.334 pF	0.201 pF
Signal degradation ^1^	−12.053 dB	−2.738 dB	−1.734 dB

^1^ Signal degradation is calculated by Equation (17) with 0.9 pF of sensor capacitance.

**Table 3:** Post-layout simulated and measured results of PGA.

	Post-Layout Simulation		Measurement	
Program Code	0 dB Gain	5 dB Gain	0 dB Gain	5 dB Gain
Sensitivity	−38.60 dBV	−34.13 dBV	−39.11 dBV	−33.76 dBV
Measured gain	−0.6 dB	+3.87 dB	−1.11 dB	+4.24 dB
Total noise level (A) ^1^	−103.84 dBV	−102.11 dBV	−106.64 dBV	−101.86 dBV
SNR (A) ^1^	65.24 dBA	67.97 dBA	66.08 dBA	68.23 dBA

^1^ (A) denotes A-weighted.

## Data Availability

Not applicable.
